# A Voxel-by-Voxel Comparison of Deformable Vector Fields Obtained by Three Deformable Image Registration Algorithms Applied to 4DCT Lung Studies

**DOI:** 10.3389/fonc.2015.00017

**Published:** 2015-02-04

**Authors:** Mirek Fatyga, Nesrin Dogan, Elizabeth Weiss, William C. Sleeman, Baoshe Zhang, William J. Lehman, Jeffrey F. Williamson, Krishni Wijesooriya, Gary E. Christensen

**Affiliations:** ^1^Department of Radiation Oncology, Virginia Commonwealth University Medical Center, Richmond, VA, USA; ^2^Department of Radiation Oncology, University of Virginia Health Systems, Charlottesville, VA, USA; ^3^Department of Electrical and Computer Engineering, The University of Iowa, Iowa City, IA, USA

**Keywords:** deformable image registration, deformable dose addition

## Abstract

**Background:** Commonly used methods of assessing the accuracy of deformable image registration (DIR) rely on image segmentation or landmark selection. These methods are very labor intensive and thus limited to relatively small number of image pairs. The direct voxel-by-voxel comparison can be automated to examine fluctuations in DIR quality on a long series of image pairs.

**Methods:** A voxel-by-voxel comparison of three DIR algorithms applied to lung patients is presented. Registrations are compared by comparing volume histograms formed both with individual DIR maps and with a voxel-by-voxel subtraction of the two maps. When two DIR maps agree one concludes that both maps are interchangeable in treatment planning applications, though one cannot conclude that either one agrees with the ground truth. If two DIR maps significantly disagree one concludes that at least one of the maps deviates from the ground truth. We use the method to compare 3 DIR algorithms applied to peak inhale-peak exhale registrations of 4DFBCT data obtained from 13 patients.

**Results:** All three algorithms appear to be nearly equivalent when compared using DICE similarity coefficients. A comparison based on Jacobian volume histograms shows that all three algorithms measure changes in total volume of the lungs with reasonable accuracy, but show large differences in the variance of Jacobian distribution on contoured structures. Analysis of voxel-by-voxel subtraction of DIR maps shows differences between algorithms that exceed a centimeter for some registrations.

**Conclusion:** Deformation maps produced by DIR algorithms must be treated as mathematical approximations of physical tissue deformation that are not self-consistent and may thus be useful only in applications for which they have been specifically validated. The three algorithms tested in this work perform fairly robustly for the task of contour propagation, but produce potentially unreliable results for the task of DVH accumulation or measurement of local volume change. Performance of DIR algorithms varies significantly from one image pair to the next hence validation efforts, which are exhaustive but performed on a small number of image pairs may not reflect the performance of the same algorithm in practical clinical situations. Such efforts should be supplemented by validation based on a longer series of images of clinical quality.

## Introduction

Finding verification and quality assurance methods for deformable image registration (DIR) algorithms remains one of the obstacles to their routine application in clinical treatment planning or radiation therapy research. The most reliable, though very labor intensive, validation methods compare DIR results to a reasonable approximation of the ground truth by using expert-delineated control points or image segmentation (1–8). These methods are limited to relatively few image pairs, as delineation of control points or image segmentation require a significant investment of time by skilled workers. Contemporary research protocols provide large numbers of images of varying quality, which presents an opportunity to study the behavior of DIR algorithms in more clinically realistic situations. Large numbers of images cannot be easily segmented; however, hence creating a need for automated analysis methods that may be less accurate but are applicable to sparsely segmented image sets. The analysis method, which is utilized in this paper, evaluates properties of DIR algorithms by comparing the behavior of two algorithms at a time (pairwise comparison) on the same anatomy. Conclusions that can be drawn from such comparisons are less definitive than conclusions drawn from comparisons to the ground truth, but the method can be readily applied to large data sets. If two algorithms consistently produce identical or similar deformation maps when applied to the same anatomy one concludes that both are interchangeable, though one cannot conclude that algorithms agree with the ground truth as both could be producing similar though erroneous results on image artifacts. Conversely, a finding of significant discrepancy between two algorithms shows that *at least one* of the algorithms deviates significantly from the ground truth as they cannot both be simultaneously correct on the same anatomy. This method of analysis cannot pick an algorithm, which is most consistent with the ground truth, but it can assess interchangeability of algorithms and stability of DIR solutions when image quality varies.

**Figure 1:**
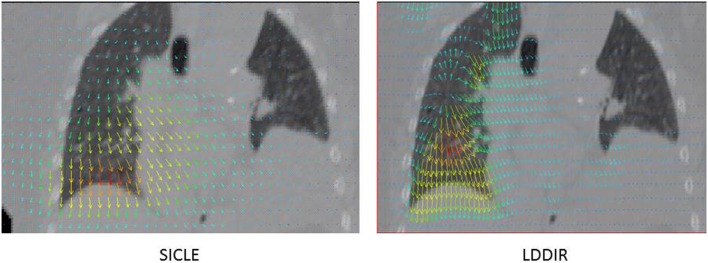
**Example of visualization of DIR results obtained with two algorithms on the same anatomy**. SICLE algorithm (9) on the left hand side and LDDIR (3) algorithm on the right hand side.

Even cursory inspection of visualizations of DIR results reveals that some algorithms can produce registration features that are physically impossible. An example of such a defect is shown in Figure 1, where registrations of peak exhale to peak inhale phases of a 4DFBCT data by two algorithms are visualized side by side. The algorithm on the left is small deformation inverse-consistent linear elastic (SICLE) image registration algorithm (9), while the algorithm on the right is a large deformation diffeomorphic image registration algorithm (LDDIR) (3). The SICLE algorithm enforces inverse consistency and is, by construction, limited to small deformation displacements, whereas the algorithm on the right is significantly less constrained. Since the defect observed in LDDIR is entirely contained within the volume of the lung it would not significantly affect deformation of the lung contour. The same defect may affect deformable dose accumulation, however, provided that defective registration occurs in a region of high dose gradient.

Results of DIR are quantified as a vector field called the deformable vector field (DVF). If one interprets DVF literally, as a measure of flow of tissue, then the DVF field contains information about local changes in the density of tissue as well as gross displacement of tissue due to anatomic change. The local change in tissue density can be assessed by a Jacobian of DVF field, while length and direction of DVF vectors can be used to assess changes in the shape of lungs due to respiratory motion. We compare two algorithms by comparing volume histograms of a Jacobian of each DVF (Jacobian volume histograms, JVHs), and volume histograms of the length of the vector difference of two DVF vectors at each voxel (spatial discrepancy volume histograms, SDVH). Volume histograms are built on contoured structures belonging to one image (target image). If the method is applied to a long series of related images, only one of the images in the series needs to be segmented.

The vector difference of two DVF vectors (spatial discrepancy, SD) is a measure of a distance between dose lookup points during the process of deformable dose accumulation by the two algorithms. If one observes large values of SD on a large portion of the volume of an organ, one may conclude that there is a potential for a discrepancy between the two algorithms during the process of deformable DVH accumulation. This potential may not always be realized for a given patient, depending on the distribution of dose gradients in this particular case, but the discrepancy indicates that two algorithms should not be used interchangeably for the purpose of treatment planning without independent dosimetric validation.

Since all images used in this work were fully segmented by a physician we were able to apply conventional contour based validation methods to the same image set. By comparing conclusions obtained by two types of analysis one can gage whether conventional segmentation based analysis methods are sufficient to detect differences between algorithms.

## Materials and Methods

### Data sets

We used 4DCT data obtained from 13 lung patients acquired at M.D.Anderson Cancer Center under IRB approval. Each 4D CT image set consisted of 10 three-dimensional image sets at discrete respiratory phases. CT images consisted of a range of number of slices 56–87, each of thickness 0.25 cm, and a square field of view of 50 cm with 512 × 512 axial resolution. The same data set has been previously used at our institution in a validation study of a large deformation diffeomorphic image registration algorithm (3). All images were processed to remove features outside of patient’s skin and image segmentation was performed in preparation for the prior study (3). Following organs were contoured by a physician: gross tumor volume (GTV), esophagus, heart, left lung, right lung, and cord.

### Deformable image registration algorithms

We present pairwise comparison of three algorithms: large deformation diffeomorphic image registration algorithm (LDDIR), which was previously used in Ref (3), SICLE image registration algorithm (9), and the ITK diffeomorphic demons algorithm (ITKDD) as implemented in the ITK package (www.itk.org). These three image registration algorithms represent three classes of image registration algorithms based on the complexity of the transformations they can accommodate. The LDDIR and ITKDD algorithms accommodate correspondence maps that can be large and curved, while the SICLE algorithm can only accommodate small deformation correspondence maps. The transformation or correspondence map is represented by a 4D vector field (3 spatial dimensions plus time) in LDDIR, a 3D vector field (spatial only) in ITKDD and by a low frequency 3D Fourier series in SICLE. The number of parameters of the transformation corresponds to the number of degrees of freedom (DOF) for representing the transformation. The LDDIR has the most DOF and is able to represent more complex transformations than the other two algorithms; the ITKDD has the second most DOF and can represent more complex transformations than the SICLE algorithm. For a 256 × 256 × 256 image, the LDDIR has 3 × 10 × 256 × 256 × 256 = 5 × 10^8^ DOF where there is a 3 vector at each voxel location and 10 time points, the ITKDD has 3 × 256 × 256 × 256 = 5 × 10^7^ DOF, and the SICLE has 3 × 2 × 10 × 10 × 10 = 6000 DOF where there are 3 complex coefficients for 10 harmonics in each of the 3 coordinate dimensions. The true DOF for the LDDIR and ITKDD are less than these totals due to the regularization used to impose spatial correlation between neighboring voxels. The regularization is used to ensure that the transformations are smooth and diffeomorphic. The LDDIR uses a viscous fluid regularization and the ITKDD uses Gaussian smoothing. The SICLE algorithm is diffeomorphic provided that the Jacobian of the transformation at each point is positive. The spatial correlation in SICLE algorithm is imposed by the Fourier series basis and a linear elastic constraint. The SICLE algorithm jointly estimates the forward and reverse transformation between two images being registered while minimizing the inverse consistency error. The inverse consistency error is the difference between the forward transformation and the inverse of the reverse transformation and vice versa. One expects that properties of SICLE algorithm make it less prone to defects shown in Figure 1, but may also potentially limit its utility in situations when large deformations are present.

### Registrations and software infrastructure

We used software infrastructure, which was developed at our institution under the name of research computing framework (RCF) (10). The software consists of a C++ library, which combines data analysis algorithms, data management features, and application building environment, which is specifically designed for the task of data processing automation on large data sets. RCF package was used to construct DVF generator, which retrieves images from our databases, preprocesses images if necessary, implements image format conversion as required by individual algorithms, invokes an algorithm on preprocessed images, post-processes DVF result if needed, and writes the final result into a database. This application was used to perform registrations between peak inhale and peak exhale respiratory phases using SICLE and ITKDD algorithms, while pre-existing LDDIR data (3) were used during data analysis. Data analysis application was written within RCF package, using data processing algorithms developed within the package.

### Data analysis algorithms

The data analysis application was built using software contained within the RCF library, most of which was developed in-house. Image segmentation contours are converted into volume bitmaps. Volume bitmaps are deformed by DVF maps volumetrically using nearest-neighbor interpolation. To create SD distributions, DVF vectors are subtracted at each voxel and stored as a new vector field. The Jacobian of a DVF map is computed at each voxel using an immediate (one voxel wide) neighborhood as a base for the calculation of derivatives, while the Jacobian in voxels located at the very edge of the volume is set to unity. Volumes are projected on segmentation bitmaps using trilinear interpolation and projections are used to build volume histograms. The DICE similarity coefficient is calculated according to the following formula (11):

$$\mathrm{DICE}=\frac{2|V_{\mathrm{deformed}}\cap V_{\mathrm{observer}}|}{\left|V_{\mathrm{deformed}}|+|V_{\mathrm{observer}}\right|}$$

which implies that DICE = 1 when volumes are perfectly matched and DICE = 0 when volumes do not overlap at all. The Jacobian determinant is computed with addition of unity matrix (12), which means that, if interpreted literally as flow of tissue, the value of the Jacobian is a direct measure of local volume change at each voxel:

$${\mathrm{Vol}}_{\mathrm{inhale}}^{\mathrm{voxel}}=|J|*{\mathrm{Vol}}_{\mathrm{exhale}}^{\mathrm{voxel}}$$

Consequently, |*J*| > 1 implies local volume expansion and |*J*| < 1 implies local volume contraction.

The vector difference between two DVF fields (SD) is computed at each voxel and the resulting SD volume is used to form SDVH.

Student’s *t*-test (Microsoft Excel) is used where applicable to assess statistical significance of differences or agreements between results, which are produced by DIR algorithms. In cases of expected discrepancy between two data sets, we use a criterion of *p* < 0.05 to claim statistical significance. In cases of expected agreement, we use a criterion of *p* > 0.05 to claim statistical significance.

## Results

### DICE similarity coefficients

**Figure 2:**
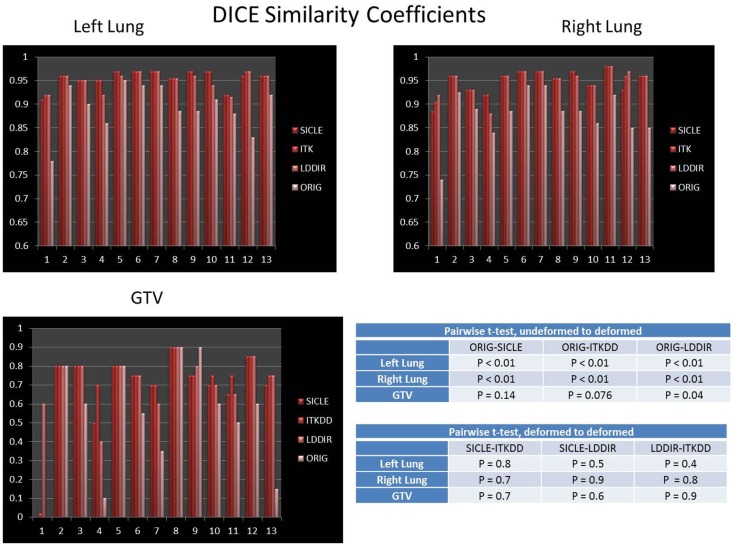
**DICE similarity coefficient prior to and post-deformation for both lungs and GTV**. Tables show *p*-values for pairwise *t*-test of differences between undeformed DICE and deformed DICE, and pairwise *t*-test for agreement between deformed DICE generated by three algorithms. The difference between DICE prior to deformation and DICE post-deformation is statistically significant in both lungs (*p* < 0.01), the post-deformation DICE agreement among three algorithms is also statistically significant in lungs (*p* > 0.4). The improvement in DICE for GTV has marginal statistical significance, with *p*-values in the range of 0.05–0.15.

DICE similarity coefficients for GTV and both lungs are shown in Figure 2. The bar labeled as “ORIG” corresponds to the DICE before warping. One notes that all three algorithms noticeably improve DICE similarity. The difference between pre- and post-deformation DICE is statistically significant for all three algorithms (*p* < 0.01). Post-deformation DICE is similar for all three algorithms particularly for larger structures like lungs (*p* > 0.5). Smaller structures, like GTV, show greater differentiation between algorithms (*p* = 0.05–0.15). Post-deformation DICE similarity for larger structures (lungs) is generally better than 90%, which is usually considered to be an excellent agreement that validates an algorithm. One should note, however, that the agreement would appear quite good (better than 80%) if no warping was done. High similarity coefficient prior to warping reflects the fact that large structures are mostly immobile, except for a relatively small portion near the diaphragm. Based on data shown in Figure 2 alone one could conclude that the three algorithms are nearly equivalent, with occasional discrepancy in more difficult images.

### Jacobian analysis

#### Mean of Jacobian volume histogram

If one takes the DVF map literally, as describing the actual flow of tissue due to anatomical change represented by the two images, one would interpret the Jacobian at each voxel as a local measure of the fractional volume change. By this interpretation, the mean value of a Jacobian distribution taken over an anatomical structure should represent the fractional volume change for the whole structure:

$$\frac{V_{\mathrm{inhale}}}{V_{\mathrm{exhale}}}=\frac{1}{V_{\mathrm{exhale}}}\sum_{i=1}^{N}v_{i}J_{i}$$

**Figure 3:**
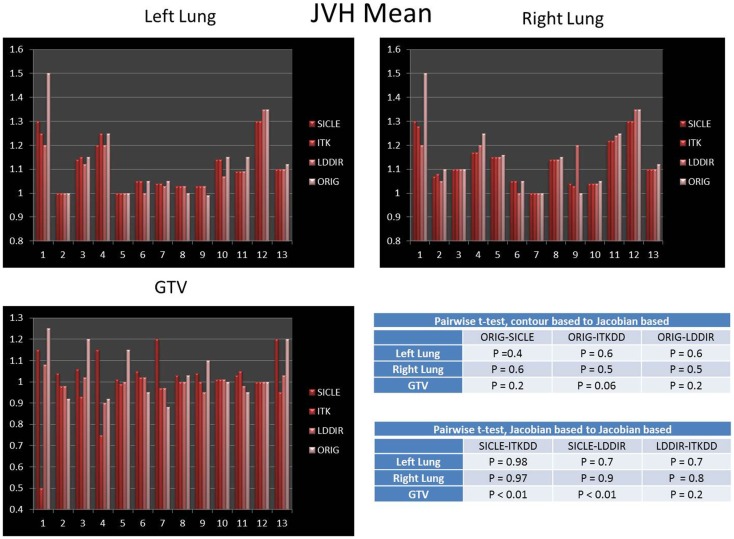
**Mean of Jacobian volume histogram for both lungs and GTV**. Tables show *p*-values for pairwise *t*-test of agreement between contour-based volume change and mean JVH, and pairwise *t*-test for agreement between mean JVH generated by three algorithms. Note that only lungs undergo significant change in volume. The agreement between contour based prediction of total volume change and mean JVH prediction is statistically significant for all pairwise comparisons in lungs (*p* > 0.4) and not significant in the GTV.

The mean of JVH computed on both lungs and the GTV is shown in Figure 3, presented together with the volume change obtained from contours drawn by a human observer. The agreement between three algorithms and actual volume change is statistically significant (*p* > 0.4) for lungs. Greater random discrepancies can be seen in smaller structures like GTV, though one should note that only lungs undergo significant volume change due to breathing. Average Jacobian normalized to the actual volume change is shown in Table 1 for all structures.

**Table 1:** **Ratio of average Jacobian to actual volume change**.

	SICLE	LDDIR	ITKDD
Left lung	1.0 ± 0.12	0.99 ± 0.12	1.0 ± 0.12
Right lung	1.0 ± 0.11	0.98 ± 0.11	1.0 ± 0.11
Heart	0.99 ± 0.05	0.94 ± 0.05	0.99 ± 0.05
Esophagus	1.03 ± 0.1	1.0 ± 0.11	1.02 ± 0.1
GTV	1.0 ± 0.11	0.96 ± 0.09	0.89 ± 0.24

*Error represents standard deviation of the ratio, computed over 13 patients*.

#### Standard deviation of Jacobian distribution

**Figure 4:**
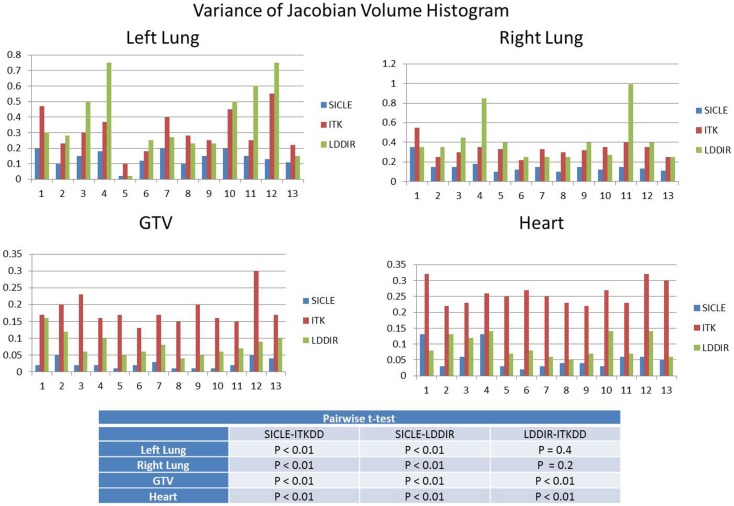
**Standard deviation of Jacobian volume histogram for both lungs, GTV, and heart**. Table shows results of pairwise *t*-test analysis to assess if differences are statistically significant. Results indicate statistical significance for all pairings in GTV and heart (*p* < 0.01), statistical significance for SICLE–ITKDD and SICLE–LDDIR pairings in lungs (*p* < 0.01), while differences between ITKDD and LDDIR are not statistically significant in either lung (*p* > 0.1).

A clear and statistically significant difference between the three algorithms can be seen when one computes standard deviation of JVH. An example of such a comparison is shown in Figure 4, and results are summarized for all structures in Table 2. The JVH variance is the smallest for SICLE on all structures. In the interior of both lungs differences between SICLE and the other two algorithms are statistically significant (*p* < 0.01), but the difference between LDDIR and ITKDD is not. For “solid” structures (like heart or GTV), ITKDD shows the largest variance by a wide margin, while LDDIR is closer to SICLE. Differences between algorithms are statistically significant (*p* < 0.01).

**Table 2:** **Standard deviation of differential Jacobian volume histogram averaged over 13 patients**.

	SICLE	LDDIR	ITKDD
Left lung	0.13 ± 0.05	0.36 ± 0.22	0.31 ± 0.13
Right lung	0.16 ± 0.06	0.41 ± 0.23	0.32 ± 0.09
Heart	0.06 ± 0.04	0.1 ± 0.03	0.27 ± 0.04
Esophagus	0.08 ± 0.05	0.15 ± 0.08	0.19 ± 0.03
GTV	0.026 ± 0.011	0.08 ± 0.03	0.19 ± 0.05

*Error represents standard deviation of the average, computed over 13 patients*.

#### Extremes of Jacobian distribution

Extremes of Jacobian distribution refer to Jacobian values, which are unlikely to represent actual physical change in tissue or, in extreme cases, are physically impossible. Negative Jacobian values are best known examples of unphysical behavior. For the purpose of this discussion, we assume that any local volume change, which is greater than a factor of two (compression or expansion), does not correspond to real change in tissue. To estimate the prevalence of extreme Jacobian values one analyzes tails of differential JVH and computes fractional volume boundaries, *J_X_*, where *X* corresponds to the fraction of the volume. In the high Jacobian tail, *J_X_* corresponds to the minimum Jacobian in the sub-volume, while in the low Jacobian tail, *J_X_* corresponds to the largest Jacobian in the sub-volume. Table 3 summarizes Jacobian volume boundaries for *X* = 2.5%. One observes that two of the three algorithms show volume boundaries approaching factor of two in volume change in both lungs, but none of the algorithms approaches these limits in other organs. One can thus observe that non-physical Jacobian values do occur, particularly in the lungs, but they affect a relatively small fraction of organ volume.

**Table 3:** **Two sided 2.5% volume boundary of differential Jacobian volume histogram for 13 patients**.

	2.5% Left margin			2.5% Right margin		
	LDDIR	SICLE	ITKDD	LDDIR	SICLE	ITKDD
Lt lung	0.57 ± 0.24	0.87 ± 0.1	0.59 ± 0.16	2.1 ± 0.73	1.33 ± 0.2	1.85 ± 0.4
Rt lung	0.5 ± 0.19	0.82 ± 0.1	0.57 ± 0.11	2.2 ± 0.85	1.39 ± 0.3	1.92 ± 0.28
Heart	0.73 ± 0.1	0.86 ± 0.1	0.51 ± 0.05	1.2 ± 0.05	1.14 ± 0.1	1.6 ± 0.11
Esophagus	0.71 ± 0.1	0.76 ± 0.3	0.64 ± 0.06	1.25 ± 0.15	1.1 ± 0.03	1.41 ± 0.08
GTV	0.85 ± 0.1	1.0 ± 0.04	0.56 ± 0.19	1.18 ± 0.1	1.16 ± 0.1	1.3 ± 0.22

*For each structure mean value and standard deviation computed over 13 patients are shown*.

### Spatial discrepancy

**Figure 5:**
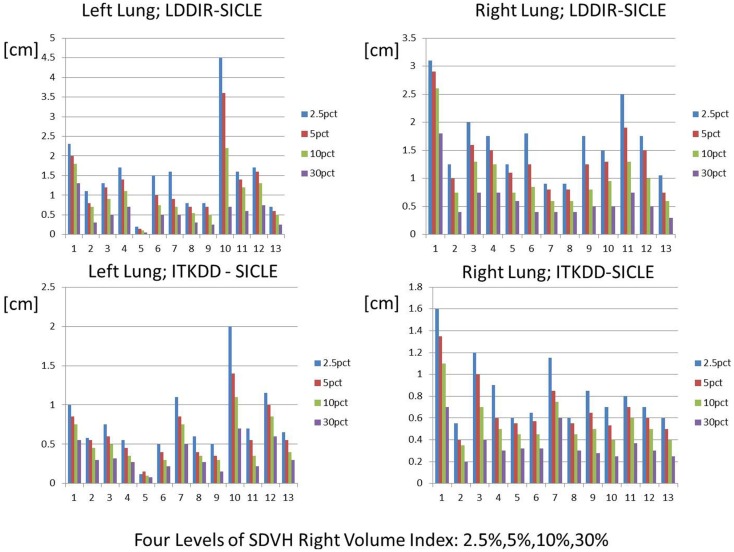
**Spatial discrepancy boundaries for a series of volume fractions in both lungs**. Data for other structures are summarized in Tables 4 and 5.

An example of SD summary plot for a lungs and GTV is shown in Figure 5, while results for all structures are summarized in Tables 4 and 5. In Figure 5, we show a series of fractional volume boundaries, (SD)*_X_* which are computed on a differential SDVH. The variable *X* identifies the percentage of structure volume on the high side of the volume histogram, and the value of (SD)*_X_* shows the smallest SD in this sub-volume. Each bar in the plot represents one value of *X* which varies from 2.5 to 30%. One observes that (SD)*_X_* values vary significantly among image pairs. For some image pairs one obtains a reasonably good agreement with SD in a sub-centimeter range, while for others one observes discrepancies in the multi-centimeter range.

**Table 4:** **2.5% volume boundary of SDVH for 13 patients**.

	SICLE–LDDIR			SICLE–ITKDD		
	Average	Min	Max	Average	Min	Max
Lt lung	1.5 ± 1.0	0.16	4.6	0.76 ± 0.44	0.12	2.0
Rt lung	1.6 ± 0.7	0.9	3.1	0.86 ± 0.29	0.56	1.6
Heart	0.68 ± 0.26	0.35	1.2	0.56 ± 0.13	0.4	0.8
Esophagus	0.53 ± 0.28	0.23	1.3	0.42 ± 0.1	0.3	0.6
GTV	0.68 ± 0.53	0.19	2.2	0.56 ± 0.36	0.2	1.1

*Error cited with averages corresponds to standard deviation over 13 patients. Spatial discrepancy in units of centimeters. Min and Max correspond to minimum and maximum SDVH boundary seen in the group of 13 patients*.

**Table 5:** **35% volume boundary of SDVH for 13 patients**.

	SICLE–LDDIR			SICLE–ITKDD		
	Average	Min	Max	Average	Min	Max
Lt lung	0.49 ± 0.31	0.06	1.3	0.3 ± 0.16	0.06	0.58
Rt lung	0.57 ± 0.35	0.3	1.6	0.32 ± 0.1	0.17	0.62
Heart	0.35 ± 0.12	0.19	0.68	0.3 ± 0.08	0.2	0.5
Esophagus	0.23 ± 0.08	0.12	0.39	0.2 ± 0.08	0.1	0.4
GTV	0.52 ± 0.52	0.08	2.1	0.4 ± 0.33	0.1	1.04

*Error cited with averages corresponds to standard deviation over 13 patients. Spatial discrepancy in units of centimeters. Min and Max correspond to minimum and maximum SDVH boundary seen in the group of 13 patients*.

## Discussion

Results presented in this work indicate that evaluation of performance of DIR algorithms is a complex process, which depends on the intended practical application of an algorithm.

Based on DICE similarity alone one can conclude that all three algorithms are functionally similar, particularly when applied to larger structures like lungs. It is thus reasonable to conclude that a task of contour propagation is relatively insensitive to which DIR algorithm is used. It has already been noted in the literature that contour based similarity measures are relatively insensitive to details of algorithm performance (13).

Analysis of JVHs reveals strong algorithm dependence of the variance of JVH. Differences in JVH variance are larger in tissue (GTV, heart) than in the lung interior. A practical implication of this finding is that Jacobian of DVF cannot be used as a measure of local volume change without some form of independent validation as all three algorithms cannot be simultaneously correct on the same structure. It is interesting to note that the mean of JVH does predict total volume change reasonably accurately for all three algorithms. This observation underscores complexities of the process of DIR validation, as it is possible to create an apparent validation of an algorithm using one or more diagnostic measures (like DICE or mean JVH) while missing poor performance in other aspects of the algorithm. A practical application of Jacobian distribution has been proposed to measure changes in lung ventilation during radiation treatment and correlate these changes with dose deposition in lungs (12). Based on data presented in this work, one can conclude that such application of DIR would require a validation method, which is specifically focused on and sensitive to the correlation between Jacobian and independently measured local volume change. Analysis of extremes (tails) of Jacobian distributions is another way to illustrate practical differences between algorithms. If one assumes that a local volume change by more than a factor of two is not physically likely, then unphysical Jacobian is observed in approximately 5% of lung volume for two of the three algorithms (ITKDD, LDDIR), while SICLE algorithm, which limits displacement and enforces inverse consistency, produces unphysical Jacobians in sub-volumes, which are much smaller, generally below 1% of the organ volume.

The SDVH based analysis shows a potential for algorithm dependence in the process of DVH accumulation for some image pairs. Large distances between dose lookup points do not occur in every registration, but in a subset of registrations one observes multi-centimeter differences, which can produce DVH accumulation errors. This result shows that efforts to validate algorithms on small sets of image pairs are incomplete to an extent that large fluctuations in the performance of algorithms do occur when one examines a longer series of images of clinical quality. It is interesting to observe that SD measures shown in Table 4 indicate that differences between SICLE and LDDIR are systematically larger than differences between SICLE and ITKDD. It is possible, though not proven by this work that differences seen in Table 4 reflect differences in constraints that the three algorithms impose on tissue motion. As discussed in Section “Deformable Image Registration Algorithms,” SICLE imposes most stringent constraints on tissue motion (including a requirement of inverse consistency), LDDIR is least constrained, and ITKDD can be placed between the two.

A more general implication of observed differences among DIR algorithms is that such algorithms cannot be regarded as providing a faithful representation of tissue motion but rather as tools, which can provide an approximation of tissue motion that may be useful for some clinical applications, if specifically validated for this particular purpose. For instance, it is possible that an algorithm may be clinically useful for the task of contour propagation, questionable when applied to the task of dose accumulation, and entirely erroneous when applied to the task of measuring a local volume change. As a further illustration of this principle, even though JVH variances vary widely among three algorithms, the mean of Jacobian distribution reflects actual volume changes of lungs reasonably well for all three. Hence, it is quite possible that mean Jacobian may still be used as a measure of volume change for the whole structure (14), at least for lungs in this analysis, even though it is not usable as a measure of local volume change.

Volume based methods of DIR analysis could be further developed into tools for automated screening of DIR results. For example, by examining a long enough sequence of clinical quality images, possibly combined with mechanical models of the lung, one can establish volume based criteria for the magnitude of displacement, which can be considered as physiologically plausible. Areas of excessive displacement can be further convoluted with dose gradients to compute a dose accumulation error metric. Registrations of the same data set with multiple algorithms to find volumes of large disagreement, convoluted with dose gradients, is also a candidate for automated error assessment algorithms. One cannot rigorously validate a registration using such measures, but one can automatically flag registrations that are likely to be flawed. 4DCT is a particularly good candidate for developing automated DIR assessment tools because phases do not require prior rigid registration making the automated process less error prone. Development of such tools requires large data sets and lies outside of the scope of the present work.

## Conclusion

We compared three DIR algorithms using DICE contour similarity measure, JVH analysis, and SD volume histogram analysis. We found that the three algorithms appear similar when compared using DICE similarity, and show significant differences when compared by the other two methods. Our analysis shows that DIR algorithms are at best approximations of tissue motion, which may be clinically useful but must be validated specifically for each clinical task to which they are applied. Specifically, the three algorithms tested are adequate for the task of contour propagation, questionable for the task of DVH accumulation and likely not adequate for tasks of measuring local volume change. We further show that registration accuracy can vary significantly among image pairs of clinical quality, which means that validation efforts that are very detailed and quantitative but based on a limited number of image pairs may not fully reflect how an algorithm performs in practical clinical situations. In summary, this work advocates for selective, task based validation of DIR algorithms, rather than efforts to achieve comprehensive validation, which would certify an algorithm as a self-consistent description of tissue change under all circumstances.

## Conflict of Interest Statement

The authors declare that the research was conducted in the absence of any commercial or financial relationships that could be construed as a potential conflict of interest.
